# Association between oxidative balance score and thyroid function and all-cause mortality in euthyroid adults

**DOI:** 10.1038/s41598-025-90491-5

**Published:** 2025-02-25

**Authors:** Qianqian Xiao, Zhanqin Zhang, Shuman Ji, Muzi Li, Bohua Zhang, Qing Xu, Chang Xiao, Huaijin Guan, Lei Ma, Xiaopeng Mei

**Affiliations:** 1 Department of Pain Management, The First Affiliated Hospital of Xi’an Jiaotong University, Xi’an, China; 2 Department of Anesthesiology, The Second Affiliated Hospital of Xi’an Jiaotong University, Xi’an, China; 3 Department of Anesthesiology, The First Affiliated Hospital of Xi’an Jiaotong University, Xi’an, China; 4 Life Sciences and Engineering College, Northwest Minzu University, Lanzhou, China; 5 Clinical College, Xi’an Medical University, Xi’an, China

**Keywords:** Oxidative balance score, Thyrotropin, Free thyroxine, All-cause mortality, Diet, Lifestyle, Health care, Medical research

## Abstract

Abnormal fluctuations in thyroid function within the reference range were strongly associated with increased all-cause mortality. This study aimed to analyze the association between oxidative balance score (OBS) and free thyroxine (FT4) and thyrotropin (TSH) in euthyroid adults, as well as their interrelationships with mortality. 5727 euthyroid adults were selected from the National Health and Nutrition Examination Survey (NHANES). Weighted linear regression investigated the potential association of OBS with FT4 and TSH. In addition, COX proportional hazard models and restricted cubic spline (RCS) were used to investigate the association between OBS, FT4, TSH, and all-cause mortality. The results showed that OBS was negatively associated with serum FT4 concentrations in euthyroid adults (− 2.95%, 95% CI − 5.16%, − 0.92%). Additionally, the all-cause mortality rate was significantly higher in the fourth quartile (Q4) of FT4 compared to the first quartile (Q1) (HR 1.40, 95% CI 1.07–1.85). In the fourth quartile of OBS, the all-cause mortality rate was 31% lower than in Q1 (HR 0.69, 95% CI 0.52–0.92). Mediation analyses indicated that FT4 partially mediated the relationship between OBS and all-cause mortality. These results suggest a significant negative association between OBS and serum FT4, while both OBS and FT4 are strongly associated with mortality. However, the effect of OBS on serum FT4 is relatively limited, and therefore its clinical significance needs to be interpreted objectively.

**Supplementary Information:**

The online version contains supplementary material available at 10.1038/s41598-025-90491-5.

## Introduction

Thyroid hormones (TH) play a pivotal role in mammals in various fields of growth and development, neural differentiation, and energy metabolism in the cardiovascular system^1^. The hypothalamic-pituitary-thyroid axis maintains normal thyroid function by regulating the concentration of thyroxine (T4) and thyroid-stimulating hormone (TSH) in the bloodstream^1,2^. The current clinical definition of normal thyroid function is primarily based on assessing thyroid function biomarkers within the normal reference range, typically the 2.5th to 97.5th percentile^3,4^. However, this approach to defining the range has certain limitations in practice, as it fails to consider the potential risks associated with clinical outcomes entirely. Several studies have demonstrated that fluctuations in serum FT4 and TSH levels may increase the risk of atrial fibrillation^5^, cardiovascular and cerebrovascular disease^6,7^, and mortality^8^, even within the reference range for thyroid function. Indeed, The development of various thyroid disorders is thought to be related to oxidative stress^9–11^.

Oxidative stress is characterized by an imbalance between the production of reactive oxygen species and the antioxidant defense system. This results in the disturbance of REDOX signaling and could potentially lead to harm at the molecular level, ultimately contributing to the onset of different diseases^12–14^. The Oxidative Balance Score (OBS) is a tool created for evaluating the oxidative stress conditions caused by diet and lifestyle, it is calculated based on a variety of dietary (pro-oxidant and antioxidant nutrients) and lifestyle exposures (smoking, alcohol consumption, obesity, and physical activity), a higher OBS indicating antioxidants are superior to pro-oxidants^15^. Several studies have shown that a reduction in OBS is linked to a higher likelihood of developing inflammatory and chronic diseases, as well as an increased risk of mortality from all causes^16^. However, few epidemiologic studies have explored the association between OBS and serum FT4, TSH in euthyroid adults.

Therefore, this study aimed to analyze the relationship between oxidative balance score (OBS) and thyroid function biomarkers among euthyroid adults and to further explore the interrelationships between OBS, FT4, TSH, and mortality.

## Methods

### Study design

**Fig. 1:**
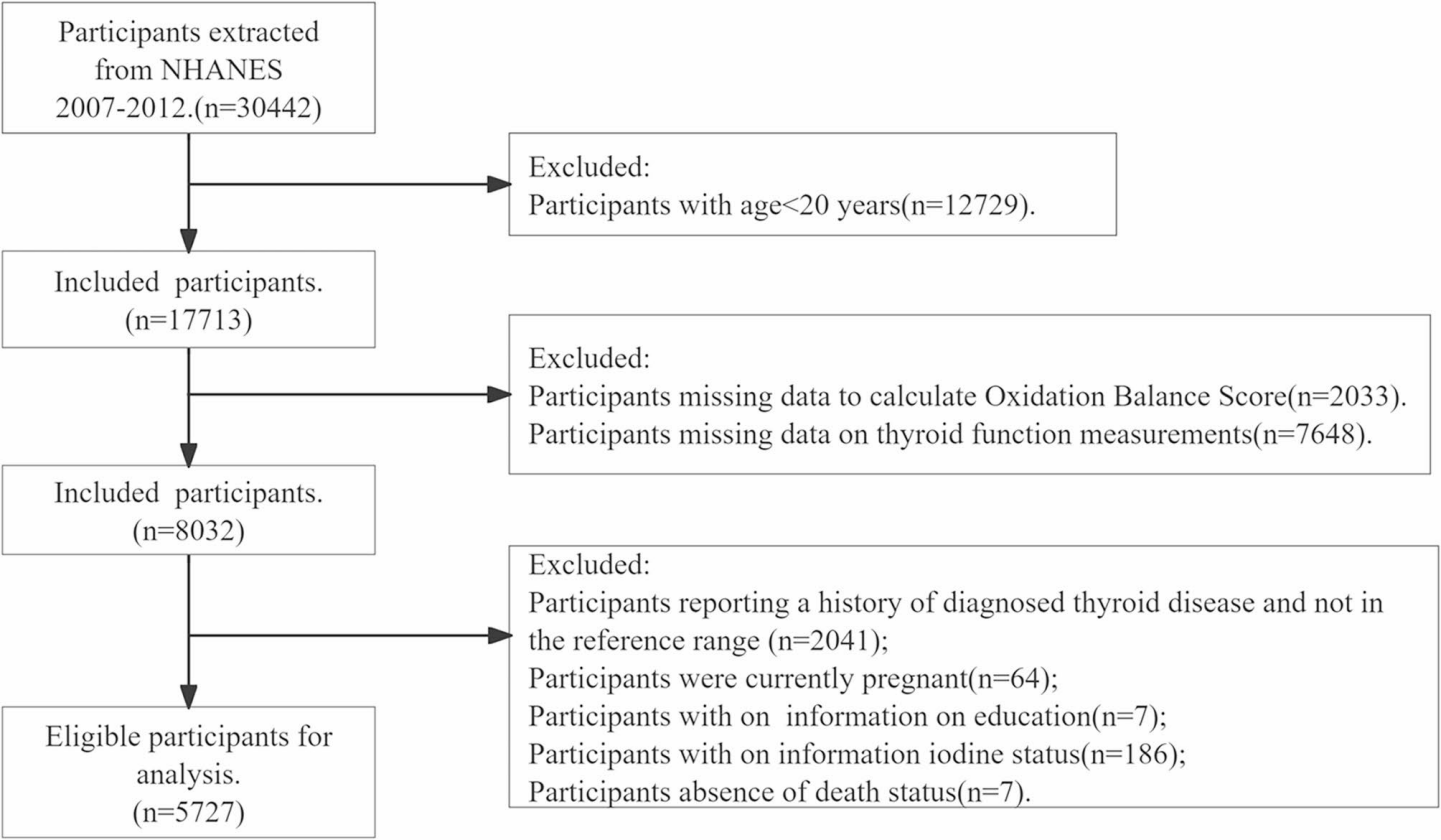
Flowchart for screening participants.

This research utilized information from the 2007–2012 National Health and Nutrition Examination Survey (NHANES), a cross-sectional study conducted by the National Centers for Disease Prevention^17^. Participants in the NHANES provided written informed consent and were approved by the National Center for Health Statistics Ethics Review Board^18^. Among the 30,442 subjects from 2007 to 2012, we excluded those who (1) were under the age of 20 (*n* = 12729), (2) had dietary (*n* = 1730) and lifestyle (303) OBS elemental deficiencies, (3) did not have thyroid function measurements (*n* = 7648), (4) reported a history of a previous diagnosis of thyroid disease (*n* = 750), (5) TSH not in the range of 0.4 to 4.5 mUI/L and FT4 concentrations not in the range of 9 to 25 pmol/L (*n* = 1291)^19,20^, (6) pregnant at the time the survey was conducted (*n* = 64), (7) missing education level (*n* = 7), (8) missing iodine status (*n* = 186), and (9) missing mortality status (*n* = 7). 5727 participants remain in the current analysis (Participant Flowchart, Fig. 1).

### Oxidative balance score

The OBS was determined using a combination of 16 dietary nutrients and four lifestyle factors, encompassing 15 antioxidant elements and five pro-oxidant components^15,21,22^. Physical activity was assessed using the NHANES-recommended metabolic equivalent of task (MET) assignment, which includes one week of “(1) vigorous work-related activity, (2) moderate work-related activity, (3) walking or biking for transportation, (4) intense leisure-time physical activity, and (5) moderate leisure-time physical activity”. The resulting score was determined based on the activity level, with 0 points for low-intensity activity, 1 for moderate-intensity activity, and 2 for high-intensity activity. Participants were categorized into heavy drinkers (≥ 15 g/day for women and ≥ 30 g/day for men), non-heavy drinkers (0–15 g/day for women and 0–30 g/day for men), and non-drinkers, receiving 0, 1, and 2 points respectively^23^. The body mass index (BMI) was categorized based on the World Health Organization’s (WHO) classification for obesity (BMI ≥ 30 kg/m^2^), overweight (BMI = 25–30 kg/m^2^), and normal weight (BMI < 25 kg/m^2^), with corresponding scores of 0–2. The remaining OBS antioxidant factors included dietary fiber, carotenoids, riboflavin, niacin, vitamin B6, total folate, vitamin B12, vitamin C, vitamin E, calcium, magnesium, zinc, copper, and selenium. The factors were divided into three categories using a three-quartile approach, and the scores for each category (1–3) ranged from 0 to 2. Higher scores indicated an increase in antioxidant levels. The remaining pro-oxidant factors included total iron and fat. Unlike antioxidants, the top tertile of pro-oxidants received a score of 0, while the bottom tertile received a score of 2. The overall OBS was calculated by adding all the components together. For more information on how each element was scored, please see (Supplementary Table 1).

### Measurement of serum FT4, TSH, and urinary iodine

Thyroid blood specimens were processed, stored and shipped to University of Washington, Seattle, WA. Detailed specimen collection and processing instructions are discussed in the NHANES Laboratory/Medical Technologists Procedures Manual (LPM) (https://www.cdc.gov/Nchs/Data/Nhanes/Public/2007/DataFiles/THYROD_E.htm). Using the Access HYPERsensitive human thyroid-stimulating hormone (hTSH) assay, which is a 3rd generation, two-site immunoenzymatic (“sandwich”) assay, to test the thyroid-stimulating hormone level. The Access FT4 assay is a two-step enzyme immunoassay. The amount of analyte in the sample is determined from a stored, multi-point calibration curve. Methodology details were described on the website (https://www.cdc.gov/nchs/nhanes/continuousnhanes/overviewlab.aspx?BeginYear=2007). Concerning the clinical practice guidelines of the American Association of Clinical Endocrinologists, we defined a participant with normal thyroid function as having a serum TSH concentration of 0.4–4.5 mUI/L and an FT4 concentration of 9–25 pmol/L^19,20^. The iodine concentration in the urine was measured using mass spectrometry with inductively coupled plasma and dynamic reaction cells^24^.

### Ascertainment of outcomes

The National Death Index (NDI) provided the mortality status and time of death for NHANES participants. The NCHS website provides corresponding methods for accessing this information (https://www.cdc.gov/nchs/data-linkage/mortality-public.htm). The International Classification of Diseases, 10th edition (ICD-10), was used to establish the cause of death. This study examined all-cause mortality as the primary outcome.

### Covariates

We incorporated variables that have been demonstrated to impact thyroid function and the risk of mortality in prior research, such as information on gender, age, race/ethnicity, level of education, status of iodine intake, and health condition. Participants’ iodine intake status was defined based on urinary iodine concentration (UIC), with UIC < 100 µg/L as iodine deficiency, 100–299 µg/L as usual, and ≥ 300 µg/L as iodine overdose^25^; the definition of Diabetes Mellitus (DM) included a physician’s report, glycosylated hemoglobin A1c (HbA1c) ≥ 6.5%, fasting blood glucose ≥ 7.0 µmol/L, or 120 min after an oral glucose tolerance test (OGTT) ≥ 11.1 mmol/L. Hyperlipidemia is defined as total cholesterol ≥ 200 mg/dL, triglycerides ≥ 150 mg/dL, LDL ≥ 130 mg/dL, or HDL < 40 mg/dL. The definition of hypertension includes self-reported diagnosis by a medical professional or an average of three consecutive measurements indicating systolic blood pressure ≥ 140 mmHg or diastolic blood pressure ≥ 90 mmHg. Cardiovascular disease was characterized by physician diagnoses obtained through personal interviews and included conditions such as coronary heart disease, angina, heart failure, heart disease, and stroke.

### Statistical analyses

In performing the statistical analyses, we followed the CDC recommendations and chose the appropriate weights for the data analyses^18^. Descriptive analysis was conducted to examine the characteristics of participants, as well as their serum FT4, TSH levels, and overall health status. Continuous variables were presented as median values, while categorical variables were expressed as percentages. Multivariate linear regression models examined the association between OBS and FT4, TSH. Due to the skewed data distribution, natural logarithm conversion is performed for OBS and FT4, TSH. The results are then expressed as percent differences^24^. We selected the covariates for adjustment based on the findings from the previous NHANES study. Variance Inflation Factor (VIF) less than 10 was considered free of multicollinearity. We used Model 1 (unadjusted), Model 2 (adjusted for age, gender, and race), and Model 3 (adjusted for age, gender, race, education, and iodine status) to explore potential associations between OBS and normal thyroid function. The continuous OBS was converted into categorical variables using quartile methods, and the P value for trends was computed. Sensitivity analyses were performed to exclude participants with serum TPOAb (> 9 IU / mL) and TgAb (> 4 IU/mL) to minimize the effect of pre-existing immune disorders of thyroid tissue. In addition, we performed a stratified analysis. A multivariate COX proportional hazard model was employed to investigate the association between OBS, FT4, TSH, and all-cause mortality. Restricted cubic spline was employed to investigate the potential for a nonlinear relationship between these three variables and all-cause mortality. When examining the relationship between OBS and all-cause mortality, we also made adjustments for diabetes, hypertension, hyperlipidemia, and cardiovascular disease. In analyzing the association between FT4, TSH, and all-cause mortality, we further refined our model by including adjustments for smoking, alcohol consumption, and BMI. To assess whether the impact of OBS and all-cause mortality were influenced by FT4 or TSH, mediation effect analyses were conducted while controlling for the covariates, including age, sex, race, education, diabetes mellitus, hypertension, hyperlipidemia, and Cardiovascular disease^26^. The R software (version 4.2.2) and EmpowerStats were utilized for all statistical analyses. Statistical significance was determined based on a two-sided P-value below 0.05.

## Results

### Participant characteristics

The median age of the participants was 44 years (95% CI 31, 57). Approximately 51.7% of the participants were male, and non-Hispanic whites accounted for a larger proportion of the participants at about 67.8%. Regarding iodine intake, 17.3% of participants had excessive intake, while 34.3% had insufficient intake. Additionally, it was found that 46.7% of the participants were smokers. Furthermore, among the participants, 11.7%, 64.9%, 30.4%, and 5.92% had diabetes, hyperlipidemia, hypertension, and cardiovascular disease respectively. Over a median follow-up period of 133 months, it was observed that approximately 9.5% of the participants died. For more detailed characteristics of the participants, please refer to (Table 1).

**Table 1:** Characteristics of study participants in NHANES 2007–2012.

Characteristic	Weighted *N* = 76,849,583 Unweighted *n* = 5727
Age	44 (31, 57)
FT_4_ (pmol/L)	10.30 (9.00, 11.60)
TSH (mUI/L)	1.59 (1.11, 2.25)
OBS	24 (18, 28)
Gender	
Male	51.7%
Female	48.3%
Race	
Mexican American	8.9%
Other Hispanic	5.6%
Non-Hispanic White	67.8%
Non-Hispanic Black	11.1%
Other Race	6.6%
Education	
Less than 9th grade	6.3%
9–11th grade	13.3%
High School Grad/GED or equivalent	80.4%
UIC	
Iodine deficient (< 100 µg/L)	34.3%
Normal (100–299 µg/L)	48.4%
Excessive iodine intake(≥ 300 µg/L)	17.3%
Smoking	
Yes	46.7%
No	53.3%
Drinking	
Yes	22.6%
No	72.3%
BMI	
Obesity	33.2%
Overweight	33.5%
Normal	33.3%
Diabetes	
Yes	11.7%
No	88.3%
Hyperlipidemia	
Yes	64.9%
No	35.1%
Hypertension	
Yes	30.4%
No	69.6%
CVD	
Yes	5.92 (%)
No	94.08 (%)

Median (IQR) or n (%).TSH, thyroid-stimulating hormone; FT4, free thyroxine; OBS, Oxidative Balance Score; BMI, body mass index; CVD, Cardiovascular disease; UIC stands for urine iodine concentration to evaluate iodine intake.

### Association of OBS with FT4 and TSH in euthyroid participants

We explored the correlation between OBS and FT4, TSH by multivariate-adjusted linear regression analysis. After adjusting all confounding variables, the results indicated a notable inverse relationship between OBS and FT4 (− 2.95%, 95% CI − 5.16%, − 0.92%). Further, we divided OBS according to the quartile method. Compared to the lowest quantile of OBS, the serum FT4 in the third quantile of OBS decreased by 2.70% (− 4.91%, − 0.41%), and that in the fourth quantile of OBS decreased by 2.59% (− 4.81%, − 0.30%) (Table 2). The observed trend of decreasing FT4 with increasing OBS was statistically significant (*p* < 0.05). To validate the robustness of these results, we also performed sensitivity analyses by excluding individuals with serum TPOAb levels exceeding 9 IU/mL and TgAb levels surpassing 4 IU/mL. This was done to minimize the influence of pre-existing immune disorders that may impact thyroid tissue. These analyses’ findings aligned with our previous results (Supplementary Table S2). In addition, we separately assessed the association of dietary OBS and lifestyle OBS with serum FT4, and the results are shown in Supplementary Table S3. The results showed a significant negative association between dietary OBS and serum FT4 after adjusting for all covariates.

(Supplementary Table S4) displays an association between OBS and FT4 in various gender subcategories. In the model adjusting for all confounding, we observed that in male participants, OBS showed a more significant negative correlation with FT4, whereas in female participants, although the two showed the same tendency to be negatively correlated, the statistical significance of this association was relatively weak. When analyzing the interaction of gender on OBS, there was no interaction of gender on OBS (P for interaction > 0.05).

**Table 2:** Adjusted percent difference (%) and 95% CI in serum thyroid function measures in relation to OBS among euthyroid participants 2007–2012.

OBS	FT4% (95% CI)	TSH % (95% CI)
Model 1		
Continuous	− 1.60 (− 3.62,0.46)*	9.14 (0.46,18.58) *
Q1	Ref	Ref
Q2	1.13 (− 1.12,3.47)	− 2.28 (− 10.67,7.15)
Q3	− 1.32 (− 3.53,0.93)	7.89 (− 1.37,18.03)
Q4	− 1.01 (− 3.17,1.18)	5.68 (− 3.17,15.35)
P for trend	0.089	0.053
Model 2		
Continuous	− 2.50 (− 4.72, − 0.46) *	5.68 (− 2.95,15.08)
Q1	Ref	Ref
Q2	0.42 (− 1.83,2.80)	− 3.84 (− 12.10,5.20)
Q3	− 2.37 (− 4.57, − 0.12) *	3.51 (− 5.38,13.24)
Q4	− 2.16 (− 4.35,0.09) *	2.80 (− 6.03,12.46)
P for trend	0.008	0.230
Model 3		
Continuous	− 2.95 (− 5.16, − 0.92) *	4.23 (− 4.50,13.76)
Q1	Ref	Ref
Q2	0.28 (− 2.05,2.56)	− 4.28 (− 12.50,4.71)
Q3	− 2.70 (− 4.91, − 0.41) *	2.33 (− 6.46,12.20)
Q4	− 2.59 (− 4.81, − 0.30) *	1.62 (− 7.32,11.43)
P for trend	0.003	0.361

FT_4_, free thyroxine; TSH, thyroid-stimulating hormone; Q, quartile; OBS, Oxidative Balance Score. Estimates are derived from a complex survey design, with three models utilized: Model 1 (unadjusted), Model 2 (adjusted for age, gender, and race), and Model 3 (adjusted for age, gender, race, education, and UIC). In the model, natural logarithm conversion was applied to adjust OBS and thyroid function indexes. The results were presented as the percentage difference in serum thyroid function per 10-unit increase in OBS. Percent differences = [e^(ln10×β)^ – 1] × 100. *P < 0.05.

### Association between OBS and all-cause mortality

**Fig. 2:**
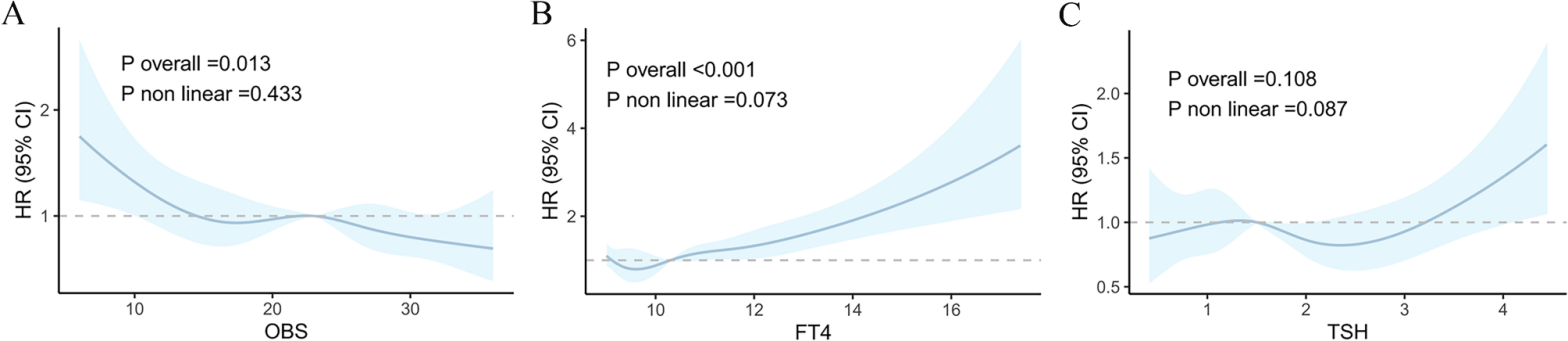
(**A**) Dose-response relationship between OBS and all-cause mortality. (**B**) Dose-response relationship between FT_4_ and all-cause mortality. (**C**) Dose-response relationship between TSH and all-cause mortality. All models were adjusted for age, gender, race, education, diabetes mellitus, hypertension, hyperlipidemia, and cardiovascular disease. The horizontal dotted line represents the hazard ratio of 1.

Our results indicated that, even after controlling for age, gender, race, education, diabetes, hyperlipidemia, hypertension, and cardiovascular disease, there was an inverse relationship between OBS and mortality. Participants with OBS in the highest percentile (75th–100th percentile) had a significant 31% lower risk of death compared to participants with OBS in the lowest percentile (0th-25th percentile). Restricted cubic spline plots provided a more visual representation (Table 3; Fig. 2A).

The associations between dietary OBS and lifestyle OBS and risk of death were assessed separately and the results are shown in Supplementary Table S5. The results showed that both dietary OBS and lifestyle OBS were negatively associated with all-cause mortality.

**Table 3:** Association between OBS and all-cause mortality.

	HR (95% CI)	*p*-value
OBS		
Continuous	0.98 (0.97,1.00)	0.008
0–25 th	Ref	
25–50th	0.88 (0.69,1.13)	0.308
50–75th	0.87 (0.67,1.13)	0.300
75–100th	0.69 (0.52,0.92)	0.012

HR, Hazard ratio; CI, confidence interval; OBS, oxidative balance score. Adjusted for age, gender, race, education, diabetes mellitus, hypertension, hyperlipidemia, and cardiovascular disease.

**Table 4:** Association between FT_4_, TSH, and all-cause mortality.

	HR (95%CI)	p-value
FT4		
Continuous	1.13 (1.07,1.19)	< 0.001
0–25th	Ref	
25–50th	0.88 (0.68,1.14)	0.340
50–75th	1.19 (0.91,1.55)	0.208
75–100th	1.40 (1.07,1.85)	0.016
P for trend		< 0.001
TSH		
Continuous	1.04 (0.93,1.16)	0.491
0–25th	Ref	
25–50th	0.98 (0.73,1.31)	0.877
50–75th	1.02 (0.76,1.35)	0.916
75–100th	0.99 (0.75,1.30)	0.921
P for trend		0.822

HR, Hazard ratio; CI, confidence interval; FT_4_, free thyroxine; TSH, thyroid-stimulating hormone; Adjusted for age, gender, race, education, smoking status, drinking status, BMI, diabetes mellitus, hypertension, hyperlipidemia, and cardiovascular disease.

### Correlation between indicators of thyroid function and all-cause mortality

In a COX proportional hazards model adjusting for age, gender, race, education, body mass index, smoking, alcohol consumption, diabetes, hyperlipidemia, hypertension, and cardiovascular disease, we observed a significant positive association between FT4 and mortality. In addition, we stratified FT4 according to quartiles, and participants with FT4 levels in the 75th–100th percentile had a substantial 40% increased risk of death compared with participants with FT4 levels in the 0th–25th percentile (*P* < 0.05). The Restricted Cubic Spline (RCS) further reinforces this finding, providing a more visual depiction of the trend (Table 4, Fig. 2B,C).

### Association between OBS, FT4, TSH, and all-cause mortality

**Fig. 3:**
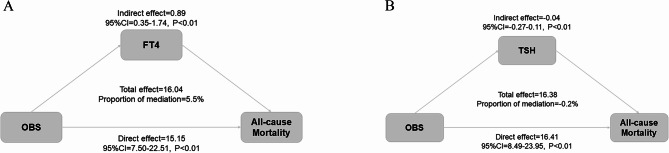
Mediation analysis for the associations between OBS and survival. FT_4_, free thyroxine; TSH, thyroid-stimulating hormone; OBS, Oxidative Balance Score. Adjusted for age, gender, race, education, diabetes mellitus, hypertension, hyperlipidemia, and cardiovascular disease.

We performed a mediation effect analysis to examine whether there was a potential association between OBS and mortality mediated by thyroid function. After comprehensive adjustment for various confounders, it was observed that FT4 partially mediated between OBS and the risk of all-cause mortality, with a mediation effect share of 5.5% (*p* < 0.01) (Fig. 3A,B).

## Discussion

Based on a nationally representative survey of U.S. adults, we found a significant negative association between OBS and serum FT4 in euthyroid adults. When assessing the association between OBS, serum FT4, and all-cause mortality, the results showed that higher serum FT4 was significantly associated with an increased risk of death, and higher OBS was associated with a lesser risk of death. Mediation analysis showed that serum FT4 had a 5.5% mediating role in the potential association between OBS and all-cause mortality.

### Negative correlation between OBS and FT4

This is the first cross-sectional study conducted in euthyroid participants to examine the association between diet and lifestyle related oxidative stress states and thyroid function. Our findings indicated a negative association between OBS and circulating FT4 levels, which remained consistent even after excluding participants with abnormal TPOAb or TgAb. Stratified analyses by gender showed that the relationship between OBS and FT4 was more statistically significant in male participants. This may be related to differences in men’s and women’s endocrine, metabolic, and immune systems^27,28^.

A cross-sectional study, encompassing individuals with normal and abnormal thyroid function, revealed a significant negative correlation between OBS and FT4^29^, consistent with our findings. However, given that abnormal fluctuations in the normal range of thyroid function-related biomarkers are also strongly associated with mortality and that oxidative stress is not only a key factor in hypothyroidism but also constitutes one of the underlying pathologic processes in hyperthyroidism^30–33^, the present study focused on a group of subjects whose thyroid function was within the reference range. We performed a sensitivity analysis (excluding patients with positive thyroid peroxidase antibodies and positive thyroglobulin antibodies) to verify more precisely the association between OBS and FT4 and TSH levels in a population with normal thyroid function. In addition, a long-term cohort study of 324 euthyroid people showed that adherence to the Mediterranean dietary pattern reduced FT3 and FT4 levels in normal thyroid functioning people^34^. Meanwhile, the Dietary Antioxidant Index (CDAI), A comprehensive assessment of six dietary antioxidants, including vitamins A, C, and E, as well as manganese (Mn), selenium (Se), and zinc (Zn), was strongly associated with a significant reduction in FT4 levels in another cross-sectional study^25^. However, when the research focused on the complementary effects of nutrients, the results differed from what was expected. A randomized controlled trial showed that participants who received zinc (Zn) and selenium (Se) supplements for 8 weeks did not experience significant changes in their thyroid hormone levels compared with placebo^35^. In addition, animal experiments showed that the combined use of vitamin E and curcumin significantly alleviated the oxidative stress state in hyperthyroid rats and effectively regulated the symptoms associated with hyperthyroidism. In contrast, no significant improvement was observed when vitamin E or curcumin was given alone^36,37^. This highlights that nutrients do not exist in isolation but are absorbed and utilized by the body as the building blocks of the overall diet. Therefore, compared with supplementing a single nutrient, a comprehensive review and optimization of individual dietary patterns may be more effective in maintaining the stability and health of thyroid function. After all, the normal operation of thyroid function is inseparable from the fine regulation and dynamic balance of various trace elements, which promote the synthesis and metabolism of thyroid hormones. In addition, the link between lifestyle and thyroid hormone levels has also been established. A cross-sectional study of 5,766 participants clearly showed that smoking habits were significantly associated with an increase in FT4 levels^38^. At the same time, most studies report a negative association between BMI and FT4 levels^39,40^. However, regarding the specific effects of alcohol consumption and physical activity on thyroid function, there are still diverging results, and no unified conclusion has been reached^41,42^.

### Antioxidant OBS reduces all-cause mortality

Prior epidemiological studies have consistently demonstrated a negative correlation between OBS and the risk of all-cause mortality, cardiovascular disease mortality, and cancer mortality^43^. Our study also found a notable link between OBS levels and all-cause mortality risk. Specifically, we observed that participants with higher OBS levels (antioxidant OBS) had a 31% lower risk of all-cause mortality than those with lower OBS levels (pro-oxidant OBS).

### Lower FT4 levels in the reference range reduce all-cause mortality

Our study focused on participants whose thyroid function was within the normal reference range and found a significant association between their serum FT4 levels and all-cause mortality. Specifically, those individuals with serum FT4 in the 75th–100th percentile had a 40% increased risk of death compared to participants with serum FT4 in the 0th–25th percentile. This finding fits with previous studies, including a survey of U.S. adults that revealed a potential link between thyroid hormones and mortality in older adults, particularly in older adults with normal thyroid function, where lower FT4 levels were significantly associated with decreased mortality^44^. Thyroid hormones are critical to the function and metabolism of all organs and tissues and, in particular, have a complex impact on the cardiovascular system, which may be one of the reasons for this association. In addition, previous studies have noted that even elevated FT4 levels within the normal reference range are associated with an increased risk of hip fracture^45^, cardiovascular disease^8^, and atrial fibrillation^46^ in adults with normal thyroid function.

Based on these results, it is particularly important to maintain serum FT4 levels in the better health range for patients treated with levothyroxine (or thyroxine), as high serum FT4 may further increase the risk of death. There is clear evidence that levothyroxine treatment is associated with a strong dose-response relationship with a significant increase in the risk of fractures^47^. Also, studies have shown that levothyroxine use is significantly associated with an elevated risk of cancer, particularly brain, skin, pancreatic, and female breast cancers^48^. Similarly, although this study was limited by a database that failed to include patients with hyperthyroidism and patients treated with levothyroxine LT4 inhibition after thyroid cancer surgery, excessive serum FT4 in these populations is similarly potentially dangerous.

### FT4 might play a role in the relationship between OBS and mortality

Thyroxine is a hormone with a wide range of physiologic effects and plays an important role in the functioning of all organ systems in the body. Studies have shown a significant correlation between high serum FT4 levels and increased mortality. Our findings suggest that OBS is strongly associated with changes in serum FT4 even within the reference range of thyroid function. FT4 mediated 5.5% of the effect of OBS on all-cause mortality. However, while there is an interesting and statistically significant relationship between OBS and serum FT4, the small effect of OBS on serum FT4 may have limited biological significance. We should objectively assess its clinical relevance.

### Strengths and limitations

Our study has important strengths on several fronts. First, we focused on the association between variation in thyroid function within the reference range and risk of death, providing the first in-depth look at the association between OBS and people with normal thyroid function. In addition, the utilization of a large, nationally representative sample enhances the generalizability of our findings. Our study has some limitations. Although the findings suggest a statistically significant association between OBS and serum FT4, the magnitude of change in this association was relatively small, probably because we included only the population with normal thyroid function. Second, although we adjusted for multiple covariates and performed sensitivity analyses to assess the robustness of our results, there may have been other confounding factors that were not considered that could have affected our results.

## Conclusion

In a nationally representative population, we found a significant negative association between OBS and serum FT4 in participants with normal thyroid function, while both OBS and FT4 were strongly associated with mortality. However, the effect of OBS on serum FT4 was relatively limited, and thus its clinical significance needs to be interpreted objectively. More studies are needed to validate our new findings.

## Electronic supplementary material

Below is the link to the electronic supplementary material.

Supplementary Material 1

## Data Availability

This study analyzed publicly available data sets. These data can be found at: https://www.cdc.gov/nchs/nhanes/index.htm.
