# Supplementary material for: Association between oxidative balance score and thyroid function and all-cause mortality in euthyroid adults
Source: Sci Rep. 2025 Feb 25;15:6817. doi: 10.1038/s41598-025-90491-5 (PMC11862123; doi:10.1038/s41598-025-90491-5)
Supplement: Supplementary file 1 — Supplementary Material 1 [file 41598_2025_90491_MOESM1_ESM.docx]

Supplementary Material

**Association between oxidative balance score and thyroid function and all-cause mortality in euthyroid adults.**

Qianqian Xiao ^1,2,†^, Zhanqin Zhang ^3,†^, Shuman Ji ^1^, Muzi Li ^4^, Bohua Zhang ^3^, Qing Xu ^1^, Chang Xiao ^3^, Huaijin Guan ^5^, Lei Ma^2^ *, Xiaopeng Mei ^1^ *

1. For more information on how each element of the OBS was scored, please see **Supplementary Table 1**.
2. To validate the robustness of these results, we also performed sensitivity analyses by excluding individuals with serum TPOAb levels exceeding 9 IU/mL and TgAb levels surpassing 4 IU/mL. This was done to minimize the influence of pre-existing immune disorders that may impact thyroid tissue. These analyses' findings aligned with our previous results (**Supplementary** **Table S2**).
3. We separately assessed the association between dietary OBS and lifestyle OBS with serum FT4, and the results are presented in **Supplementary Table S3**. The results showed a significant negative association between dietary OBS and serum FT4 after adjusting for all covariates.
4. **Supplementary** **Table S4** displays an association between OBS and FT4 in various gender subcategories. In the model adjusting for all confounding, we observed that in male participants, OBS showed a more significant negative correlation with FT4, whereas in female participants, although the two showed the same tendency to be negatively correlated, the statistical significance of this association was relatively weak. When analyzing the interaction of gender on OBS, there was no interaction of gender on OBS (P for interaction > 0.05).
5. The associations between dietary OBS and lifestyle OBS and risk of death were assessed separately and the results are shown in **Supplementary Table S5**. The results showed that both dietary OBS and lifestyle OBS were negatively associated with all-cause mortality.

| **Supplementary Table S1.** Oxidative balance score (OBS) items and score assignment | | | | |
| --- | --- | --- | --- | --- |
| OBS components | Assignment Scheme | | | |
| Dietary OBS components | |  |  |  |
| Dietary fiber (g/d) | A | 0 = low (1st tertile), 1 = intermediate (2nd tertile), 2 = high (3rd tertile) | | |
| Carotene (RE/d) | A | 0 = low (1st tertile), 1 = intermediate (2nd tertile), 2 = high (3rd tertile) | | |
| Riboflavin (mg/d) | A | 0 = low (1st tertile), 1 = intermediate (2nd tertile), 2 = high (3rd tertile) | | |
| Niacin (mg/d) | A | 0 = low (1st tertile), 1 = intermediate (2nd tertile), 2 = high (3rd tertile) | | |
| Vitamin B6 (mg/d) | A | 0 = low (1st tertile), 1 = intermediate (2nd tertile), 2 = high (3rd tertile) | | |
| Total folate (mcg/d) | A | 0 = low (1st tertile), 1 = intermediate (2nd tertile), 2 = high (3rd tertile) | | |
| Vitamin B12 (mcg/d) | A | 0 = low (1st tertile), 1 = intermediate (2nd tertile), 2 = high (3rd tertile) | | |
| Vitamin C (mg/d) | A | 0 = low (1st tertile), 1 = intermediate (2nd tertile), 2 = high (3rd tertile) | | |
| Vitamin E (ATE) (mg/d) | A | 0 = low (1st tertile), 1 = intermediate (2nd tertile), 2 = high (3rd tertile) | | |
| Calcium (mg/d) | A | 0 = low (1st tertile), 1 = intermediate (2nd tertile), 2 = high (3rd tertile) | | |
| Magnesium (mg/d) | A | 0 = low (1st tertile), 1 = intermediate (2nd tertile), 2 = high (3rd tertile) | | |
| Zinc (mg/d) | A | 0 = low (1st tertile), 1 = intermediate (2nd tertile), 2 = high (3rd tertile) | | |
| Copper (mg/d) | A | 0 = low (1st tertile), 1 = intermediate (2nd tertile), 2 = high (3rd tertile) | | |
| Selenium (mcg/d) | A | 0 = low (1st tertile), 1 = intermediate (2nd tertile), 2 = high (3rd tertile) | | |
| Total fat (g/d) | P | 0 = high (3rd tertile), 1 = intermediate (2nd tertile), 2 = low (1st tertile) | | |
| Iron (mg/d) | P | 0 = high (3rd tertile), 1 = intermediate (2nd tertile), 2 = low (1st tertile) | | |
| Lifestyle OBS components |  |  | | |
| Physical activity (MET-minute/week) | A | 0 = low (1st tertile), 1 = intermediate (2nd tertile), 2 = high (3rd tertile) | | |
| Alcohol drinking status | P | 0 = heavy drinker, 1 = mild-to-moderate drinker, 2 = non-drinker | | |
| Body mass index (kg/m2) | P | 0 = obese, 1 = overweight, 2= normal | | |
| Smoking status | P | 0 = current smoker, 1 = former smoker, 2 = never smoker | | |

OBS: oxidative balance score; A: antioxidant; P: prooxidant; RE: retinol equivalent; ATE: alpha-tocopherol equivalent; MET: metabolic equivalent.

| **Supplementary Table S2.** Sensitivity analyses. | | |
| --- | --- | --- |
| OBS | FT4% (95% CI) | TSH% (95% CI) |
| Model 1 | | |
| Continuous | -2.28(-4.50,-0.23) * | 10.41(1.39,20.50) * |
| Q1 | Ref | Ref |
| Q2 | 0.93(-1.37,3.51) | -0.23(-9.43,9.65) |
| Q3 | -1.60(-4.10,0.69) | 10.66(0.69,21.62) * |
| Q4 | -2.05(-4.28,0.23) | 5.20(-4.28,15.35) |
| P for trend | 0.008 | 0.092 |
| Model 2 | | |
| Continuous | -3.17(-5.38,-0.92) * | 6.66(-2.28,16.68) |
| Q1 | Ref | Ref |
| Q2 | 0.23(-2.05,2.80) | -2.50(-11.08,7.15) |
| Q3 | -2.50(-4.72,-0.23) * | 5.93(-3.62,16.41) |
| Q4 | -2.95(-5.16,-0.69) * | 2.09(-7.10,12.20) |
| P for trend | 0.001 | 0.34 |
| Model 3 | | |
| Continuous | -3.62(-5.81,-1.37) * | 5.93(-3.39,15.88) |
| Q1 | Ref | Ref |
| Q2 | 0.23(-2.28,2.57) | -2.50(-11.28,7.15) |
| Q3 | -2.95(-5.16,-0.46) * | 4.95(-4.72,15.61) |
| Q4 | -3.39(-5.81,-0.92) * | 1.62(-7.74,11.69) |
| P for trend | <0.001 | 0.429 |

Abbreviations: FT_4_, free thyroxine; TSH, thyroid-stimulating hormone; Q, quartile; OBS, Oxidative Balance Score. Estimates are derived from a complex survey design, with three models utilized: Model 1 (unadjusted), Model 2 (adjusted for Age, Gender, Race), and Model 3 (adjusted for Age, Gender, Race, Education, and UIC). In the model, natural logarithm conversion was applied to adjust OBS and thyroid function indexes. The results were presented as the percentage difference in serum thyroid function per 10-unit increase in OBS. Percent differences = [e^(ln10×β)^ – 1] × 100. *P < 0.05.

| **Supplementary Table S3.** Adjusted Percent Difference (%) and 95% CI in Serum Thyroid Function Measures in Relation to dietary/lifestyle OBS among euthyroid Participants 2007–2012. | | | | |
| --- | --- | --- | --- | --- |
| Variables | FT4 % (95% CI) | *P* | TSH % (95% CI) | *P* |
|  |  |  |  |  |
| Dietary OBS |  |  |  |  |
| Model 1 | -1.14 (-3.4,1.16) | 0.350 | 9.65(-0.22,17.49) | 0.062 |
| Model 2 | -2.05 (-4.28,0.23) | 0.092 | 4.71(-4.50,14.82) | 0.287 |
| Model 3 | -2.28 (-4.5,-0.23)* | 0.039 | 4.71(-4.50,14.82) | 0.432 |
| Lifestyle OBS |  |  |  |  |
| Model 1 | 0.93 (-1.83,4.00) | 0.498 | -2.28(-10.87,7.15) | 0.545 |
| Model 2 | 1.40 (-1.60,4.47) | 0.354 | -0.02(-8.78,9.57) | 0.996 |
| Model 3 | 1.39 (-1.60,4.47) | 0.373 | -2.28(-10.87,9.65) | 0.787 |

Abbreviations: FT_4_, free thyroxine; TSH, thyroid-stimulating hormone; OBS, Oxidative Balance Score. Estimates are derived from a complex survey design, with three models utilized: Model 1 (unadjusted), Model 2 (adjusted for age, gender, and race), and Model 3 (adjusted for age, gender, race, education, and UIC). In the model, natural logarithm conversion was applied to adjust OBS and thyroid function indexes. The results were presented as the percentage difference in serum thyroid function per 10-unit increase in OBS. Percent differences = [e^(ln10×β)^ – 1] × 100. *P < 0.05.

| **Supplementary Table S4.** Association between oxidative balance scores and thyroid function in euthyroid participants by gender group stratification. | | |
| --- | --- | --- |
| OBS | FT4% (95% CI) | TSH% (95% CI) |
| Male N=3066 | -4.04(-7.10,-0.69) * | 4.71(-6.67,20.23) |
| Female N=2661 | -2.73(-5.59,0.23) | 2.33(-10.87,14.82) |
| p for interaction | 0.701 | 0.671 |

Abbreviations: TSH, thyroid-stimulating hormone; FT_4_, free thyroxine; OBS, Oxidative Balance Score. Estimates are derived from a complex survey design, Age, Gender, Race, Education level, and UIC were adjusted. In the model, natural logarithm conversion was applied to adjust OBS and thyroid function indexes. The results were presented as the percentage difference in serum thyroid function per 10-unit increase in OBS. Percent differences = [e^(ln10×β)^ – 1] × 100. *P < 0.05.

| **Supplementary Table S5.** Association between dietary/lifestyle OBS with all-cause mortality. | | |
| --- | --- | --- |
| Variables | HR (95%CI) | *P* |
|  |  |  |
| Model 1 |  |  |
| Dietary OBS | 0.98 (0.97 - 0.99) | 0.001 |
| Lifestyle OBS | 0.81 (0.75 - 0.87) | <0.001 |
| Model 2 |  |  |
| Dietary OBS | 0.98 (0.97 - 0.99) | 0.005 |
| Lifestyle OBS | 0.92 (0.84 - 0.99) | 0.044 |

Abbreviations: HR: Hazard Ratio, CI: Confidence Interval; OBS, Oxidative Balance Score. Model 1 (unadjusted); Model 2 (Adjusted for Age, Gender, Race, Education, Diabetes mellitus, Hypertension, Hyperlipidemia, and Cardiovascular disease).
